# Publisher Correction: Whole-Brain Vasculature Reconstruction at the Single Capillary Level

**DOI:** 10.1038/s41598-019-42216-8

**Published:** 2019-06-14

**Authors:** Antonino Paolo Di Giovanna, Alessandro Tibo, Ludovico Silvestri, Marie Caroline Müllenbroich, Irene Costantini, Anna Letizia Allegra Mascaro, Leonardo Sacconi, Paolo Frasconi, Francesco Saverio Pavone

**Affiliations:** 1 European Laboratory for Non-Linear Spectroscopy, University of Florence, Via Nello Carrara 1, Sesto Fiorentino, 50019 Italy; 2 Department of Information Engineering (DINFO), University of Florence, Via di S. Marta 3, Florence, 50139 Italy; 3 National Institute of Optics, National Research Council, Largo Fermi 6, Florence, 50125 Italy; 4 Neuroscience Institute, National Research Council, Via Giuseppe Moruzzi 1, Pisa, 56125 Italy; 5 Department of Physics and Astronomy, University of Florence, Via Sansone 1, Sesto Fiorentino, 50019 Italy

Correction to: *Scientific Reports* 10.1038/s41598-018-30533-3, published online 22 August 2018

In the Supplementary Information file originally published with this Article, Supplementary Figures S1-S4, the Supplementary Video legends and the Supplementary Methods were omitted. These errors have been corrected in the Supplementary Information that now accompanies the Article.

