# Supplementary material for: Whole-Brain Vasculature Reconstruction at the Single Capillary Level
Source: Sci Rep. 2018 Aug 22;8:12573. doi: 10.1038/s41598-018-30533-3 (PMC6105658; doi:10.1038/s41598-018-30533-3)
Supplement: Supplementary file 1 — Supplementary information [file 41598_2018_30533_MOESM1_ESM.docx]

**WHOLE-BRAIN VASCULATURE RECONSTRUCTION AT THE SINGLE CAPILLARY LEVEL**

Antonino Paolo Di Giovanna^1^, Alessandro Tibo^2^, Ludovico Silvestri^3,1^, Marie Caroline Müllenbroich^3,1^, Irene Costantini^1^, Anna Letizia Allegra Mascaro^4,1^, Leonardo Sacconi^3,1^, Paolo Frasconi^2^, and Francesco Saverio Pavone^1,5,3^*

*email: pavone@lens.unifi.it

^1^European Laboratory for Non-linear Spectroscopy, University of Florence, Via Nello Carrara 1, Sesto Fiorentino, 50019 Italy;

^2^Department of Information Engineering (DINFO), University of Florence, Via di S. Marta 3, Florence, 50139 Italy;

^3^National Institute of Optics, National Research Council, Largo Fermi 6, Florence, 50125 Italy;

^4^Neuroscience Institute, National Research Council, Via Giuseppe Moruzzi 1, 56125 Pisa, Italy;

^5^Department of Physics and Astronomy, University of Florence, Via Sansone 1, Sesto Fiorentino, 50019, Italy

**Supplementary Figures**


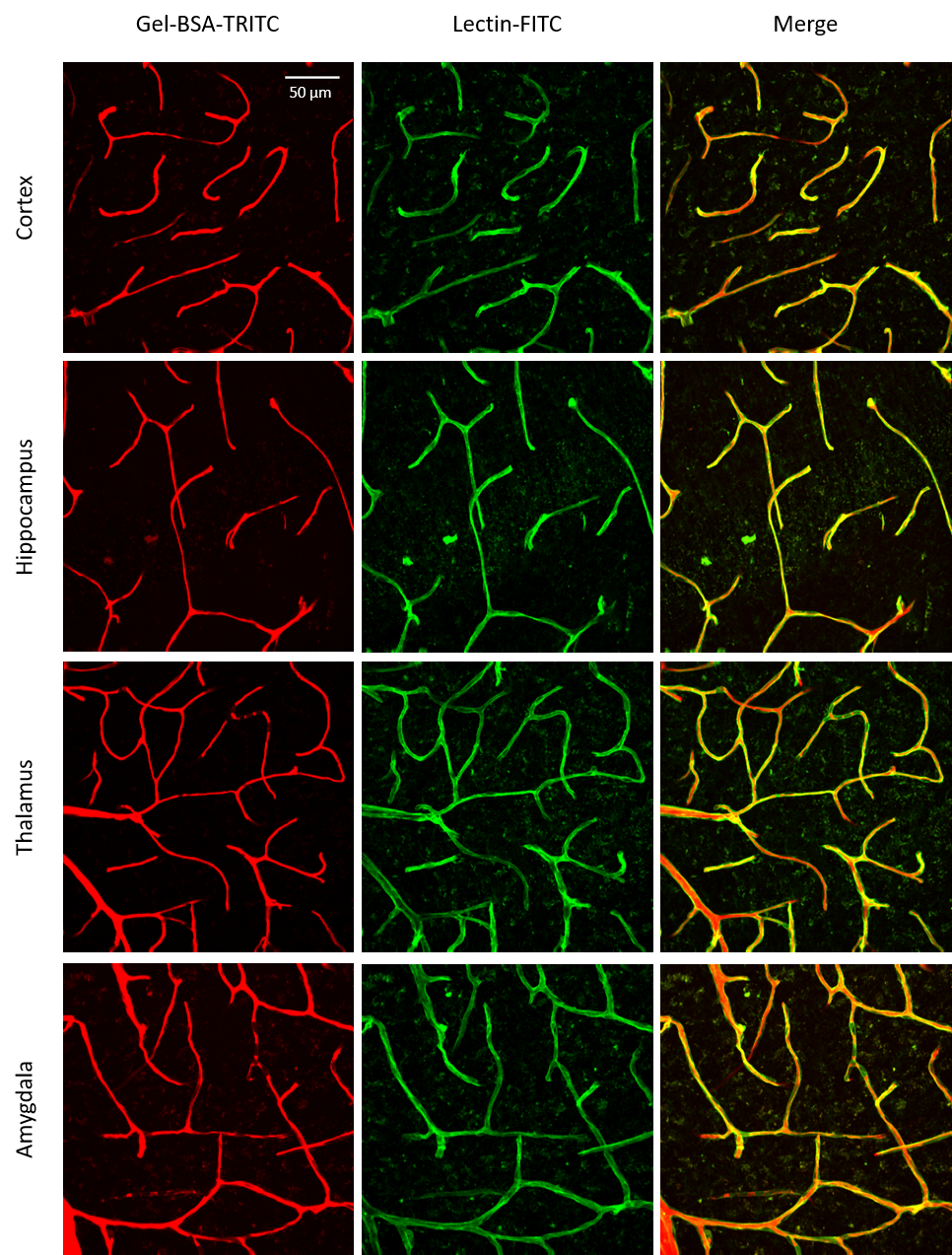


**Supplementary Figure S1. Gel perfusion efficiency.** Comparison between gel perfusion and endothelial staining revealed an efficient gel perfusion of the blood vessels in brain cortex (98.6 ± 0.1%), hippocampus (96.2 ± 0.3%), thalamus (97.4 ± 0.3%), and amygdala (95.5 ± 0.3%) (mean ± sd, n=3 mice). Imaging with TPFM, MIPs 50 µm.


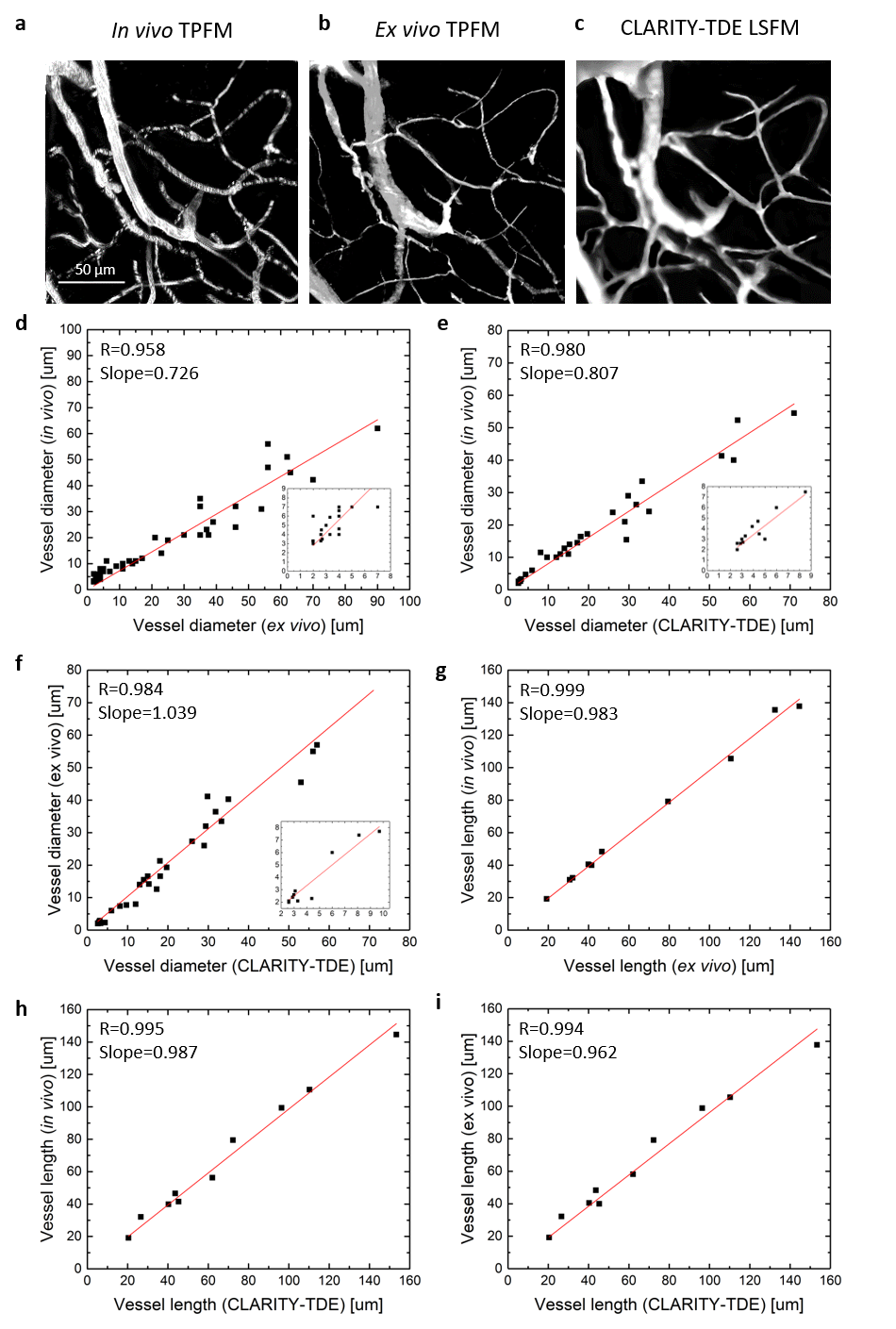


**Supplementary Figure S2. Assessment of morphological changes during brain preparation.** (**a**-**c**) A single mouse brain region of the motor cortex imaged using two-photon fluorescence microscopy (TPFM) in vivo through a cranial window (Texas-red Dextran labelling) (**a**), ex vivo TPFM (gel-BSA-FITC labelling (**b**), and light-sheet fluorescence microscopy (LSFM) after tissue clearing (**c**). (**d**-**f**) Comparison of vessel dimensions between *in vivo* and *ex vivo* TPFM (**d**), between *in vivo* TPFM and *ex vivo* LSFM on clarified sample (**e**), and between *ex vivo* TPFM and *ex vivo* LSFM (**f**). Insets show a zoom in with replotted data. R=0.946, slope=1.397 for inset in (**d**), R=0.967, slope=0.855 for inset in (**e**), R=0.950, slope=0.896 for inset in (**f**). (**g**-**i**) Comparison of vessel segment lengths between *in vivo* and *ex vivo* TPFM (**g**), *in vivo* TPFM and *ex vivo* LSFM (**h**), and *ex vivo* TPFM and *ex vivo* LSFM (**i**).


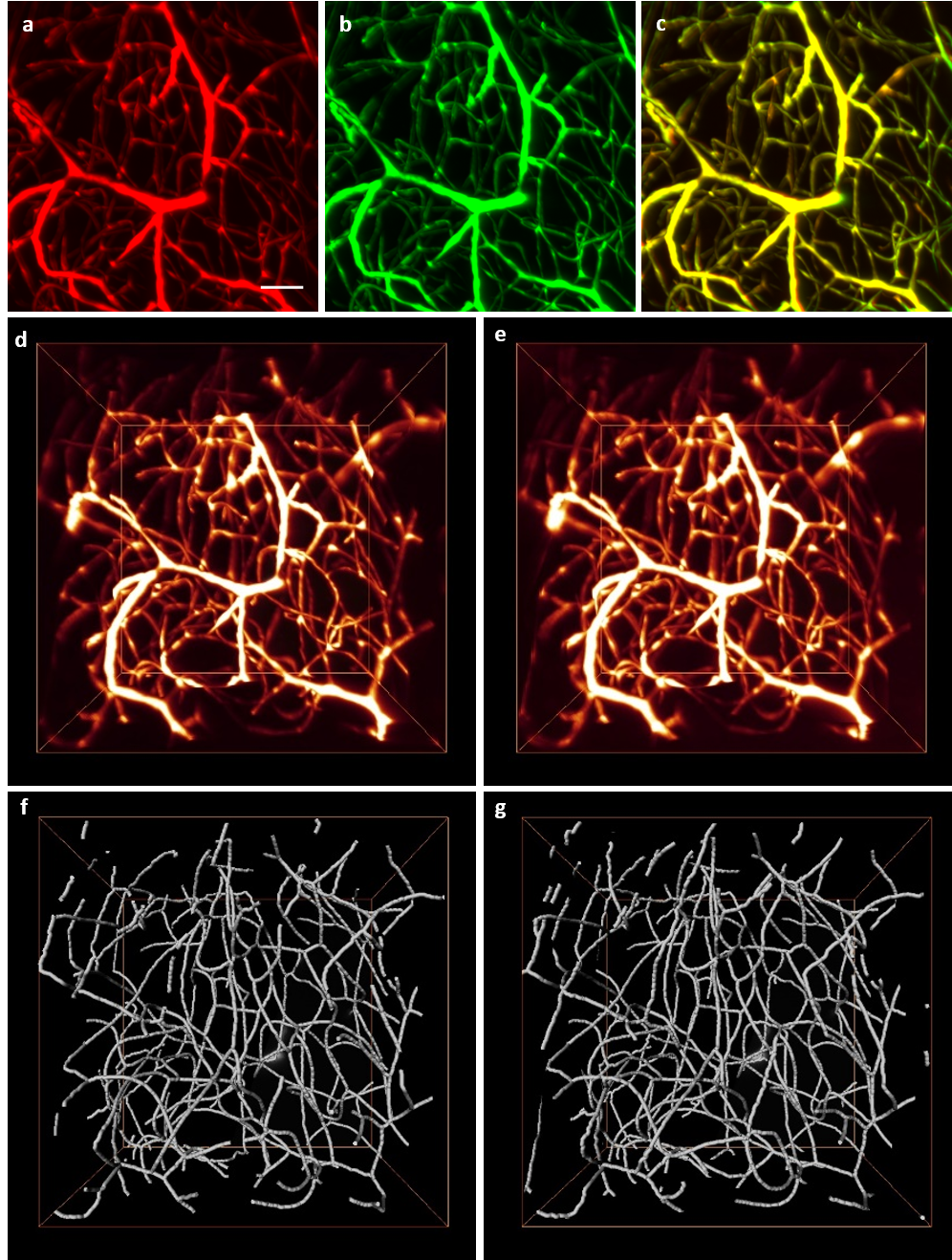


**Supplementary Figure S3. Evaluation of optical distortions.** (**a**-**c**) An internal brain area of the hippocampus was acquired through the dorsal side (**a**) and the ventral side (**b**) of the brain. Superimposition of MIPs from the two acquisition show no remarkable differences (**c**). (**d**, **e**) 3D rendering with Amira software of the above stacks. (**f**, **g**) 3D vascular tracing obtained with Amira software after image segmentation. Volume for each stack: 361×361×350 µm. Total vascular length measured with Amira software: 18. 00 mm (**f**), and 18, 37 mm (**g**).


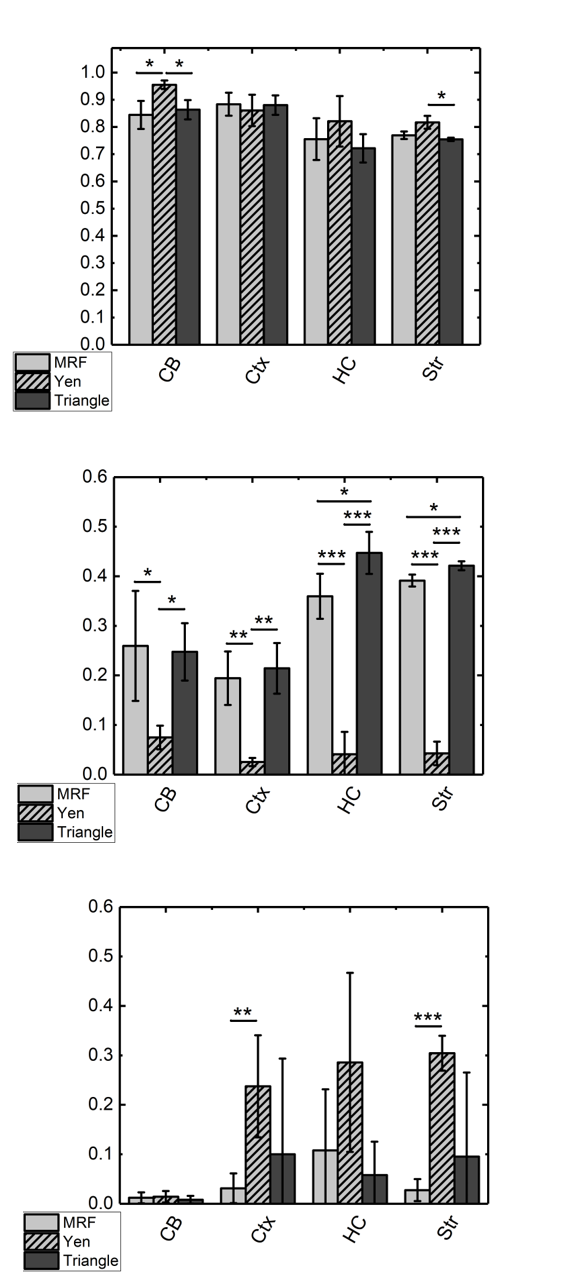


**Supplementary Figure S4. Markov random field vs simple thresholding segmentation.** Comparison between Markov random field (MRF) and simple thresholding segmentations based either on Triangle or Yen method. (**a**) True positive, (**b**) false positive and (**c**) false negative rates are reported. Simple thresholding with Yen method shows low FPR, but a significant increase of FNR is observed for cortex and striatum with respect to MRF. The Triangle method results instead in higher FPR values in internal regions such as HC and Srt. Based on these results, we assert that MRF method appears more balanced regarding FP and FN ratios. (mean ± sd, n= 4 MIPs). CB = cerebellum, Ctx = cortex, HC = hippocampus, Str = striatum.

**Supplementary Videos**

**Supplementary video 1. Whole-brain 3D rendering of the vascular network.** 3D rendering of downsampled whole-brain vasculature dataset obtained with Amira 5.3 software. Stacks were stitched using TeraStitcher software[^38^](#_ENREF_38). Orientation is indicated as R= rostral, C=caudal, D= dorsal, and V= ventral, with respect to the whole brain orientation. MCA= middle cerebral artery, ACA= anterior cerebral artery.

**Supplementary video 2. Vascular rendering of ROI from the whole-brain dataset.** 3D rendering of the vasculature from 1.3×1.3×2 mm ventral brain region at the correspondence of the right middle cerebral artery sprouting from the Circle of Willis. Image obtained with Amira 5.3 software from LSFM image stack downsampled by a factor of 2 with respect to the original resolution. Orientation is indicated as R= rostral, C= caudal, D= dorsal, and V= ventral, with respect to the whole brain orientation.

**Supplementary video 3. Neuronal rendering from *Thy1*-GFP-M mouse perfused with gel-BSA-TRITC.** 3D rendering of 1.3×1.3×2 mm from downsampled image stack showing GFP-expressing neurons under the *thy1* promoter. The *Thy1*-GFP-M brain sample was treated for vasculature staining and clearing as described. Image obtained with Amira 5.3 software from downsampled LSFM image stack. Orientation is indicated as R= rostral, C=caudal, M= medial, and L= lateral, with respect to the whole brain orientation.

**Supplementary video 4. Vascular rendering from Thy1-GFP-M mouse perfused with gel-BSA-TRITC.** 3D rendering showing blood vessels from the same portion of brain showed in supplementary video 3. Image obtained with Amira 5.3 software from downsampled LSFM image stack. Orientation is indicated as R= rostral, C=caudal, M= medial, and L= lateral, with respect to the whole brain orientation.

**Supplementary Methods**

**Automatic image segmentation**

The model was trained using an EM-like algorithm. Given a 3D image Y, the model predicts a label image X whose voxels take values on f0; 1g. MRF is a two-level hierarchical probabilistic model where in the first level (Equation 1) voxels are assumed to be mutually independent given the labels and normally distributed within each class. The second level of the model describes the prior probability on the label image (Equation 2).

| $P\left( Y \vert X,\mu,\sigma\right)=\prod_{i} \mathcal{N(}y_{i};\mu_{x_{i}},\sigma_{x_{i}})$ | (1) |
| --- | --- |

where $\mathcal{N}$ represents a gaussian distribution and *µ* = (*µ*_0,_ *µ*_1_) and$\sigma$ = ($\sigma$_0_, $\sigma$_1_) are unknown parameters which represent means and standard deviations, respectively.

| $P\left( X \vert\beta\right)=\frac{1}{Z(\beta)}e^{-\beta U(X)},U\left( X \right)=\sum_{i,j\in N_{i}} U_{ij}(x_{i},x_{j})$ | (2) |
| --- | --- |

where $\beta$ is a hyperparameter that controls the width of the NRF, $Z(\beta)$ a normalization constant and $N_{i}$ is the set of voxels which are neighbors of voxel *i*.$U_{ij}$ is a pairwise interaction energy function e.g. $U_{ij}\left( x_{i},x_{j} \right)=\frac{w_{i}j}{2}\left( 1-\delta\left( x_{i},x_{j} \right) \right)$, where $w_{i}j$ represents the inverse Euclidean distance between voxels *i* and *j* and $\delta\left( \cdot, \cdot\right)$ is the Kronecker delta.

**Two-Photon Fluorescence Microscopy Imaging**

A custom-made two-photon fluorescence microscope (TPFM) was assembled from a mode-locked Ti:Sapphire laser (Chameleon, 120 fs pulse width, 80 MHz repetition rate, Coherent, CA) coupled with a custom-made scanning system based on a pair of galvanometric mirrors (VM500+, Cambridge Technologies, MA). The laser light was focused onto the specimen using a water-immersion 20× objective lens (XLUM 20, NA 0.95, WD 2 mm, Olympus, Japan) for fixed specimens and *in vivo* measurements. For gel-BSA-FITC and lectin-FITC stained samples cleared with 47% TDE/PBS (RI 1.42), we used a tuneable 20× objective lens (Sca/e LD SC Plan-Apochromat, NA 1, WD 5.6 mm, Zeiss, Germany). Imaging in depth was performed with 2-µm z-step. The system was equipped with a motorized *xy* stage (MPC-200, Sutter Instrumente, CA) for lateral displacement of the sample and with a closed-loop piezoelectric stage (ND72Z2LAQ PIFOC objective scanning system, 2-mm travel range, Physik Instrumente, Germany) for the displacement of the objective along the *z* axis. The fluorescent light was separated from the laser optical path by a dichroic beam splitter (DM1) positioned as close as possible to the objective lens (non-de-scanning mode). A two-photon fluorescence cut-off filter (720 SP) eliminated rejected laser light. A second dichroic mirror (DM2) was used to split the two spectral components of the fluorescence signal. The fluorescence signals were filtered with 630/69 and 510/42 filters (FF1 and FF2) and collected by two orthogonal photomultiplier modules (H7422P, Hamamatsu Photonics, Japan). The instrument was controlled by custom software, written in LabView (National Instruments, TX).

**Light-sheet microscopy imaging**

Whole brains were imaged using a custom-made light-sheet microscope described in Muellenbroich et al.[^22^](#_ENREF_22) The light sheet was generated using a laser beam scanned by a galvanometric mirror (6220H, Cambridge Technology, MA); confocality was achieved by synchronizing the galvo scanner with the line read-out of the sCMOS camera (Orca Flash4.0, Hamamatsu Photonics, Japan). The laser light was provided by a diode laser (Excelsior 488, Spectra Physics) and an acousto-optic tuneable filter (AOTFnC- 400.650-TN, AA Opto-Electronic, France) was used to regulate laser power. The excitation wavelengths were λ = 561 nm for TRITC and λ = 491 nm for GFP and fluorescein. The excitation objective was a 10×, 0.3 NA Plan Fluor from Nikon, while the detection objective was a 10×, 0.6 NA Plan Apochromat from Olympus. The latter had a correction collar for the refractive index of the immersion solution, ranging from 1.33 to 1.52. The samples were placed in a quartz cuvette containing the mounting medium (63% TDE/PBS) and placed in a custom-made chamber filled with the mounting medium. The samples were mounted on a motorized *x*-, *y*-, *z*-, *ϴ*-stage (M-122.2DD and M-116.DG, Physik Instrumente, Germany), which allowed free 3-D motion and rotation. Stacks were acquired with a *z*-step of 2 µm and an *xy* resolution resulting from the setup configuration of 0.65 µm, with a field of view of 1.3×1.3 mm. The microscope was controlled via custom written LabVIEW code (National Instruments), which coordinated the galvo scanners, the rolling shutter, and the stack acquisition.
